# Dialectics of mindfulness: implications for western medicine

**DOI:** 10.1186/1747-5341-6-10

**Published:** 2011-05-17

**Authors:** Sebastian Sauer, Siobhan Lynch, Harald Walach, Niko Kohls

**Affiliations:** 1 Generation Research Program, Ludwig-Maximilians-University, Munich, Germany; 2 Peter-Schilffarth-Institute, Bad Toelz, Germany; 3 School of Social Sciences, The University of Northampton, Northampton, UK; 4 Institute of Psychiatry, King's College London, London, UK; 5 Institute of Transcultural Health Studies, European University Viadrina, Frankfurt (Oder), Germany; 6 Brain, Mind and Healing Program, Samueli Institute, Alexandria, USA

## Abstract

Mindfulness as a clinical and nonclinical intervention for a variety of symptoms has recently received a substantial amount of interest. Although the application of mindfulness appears straightforward and its effectiveness is well supported, the concept may easily be misunderstood. This misunderstanding may severely limit the benefit of mindfulness-based interventions. It is therefore necessary to understand that the characteristics of mindfulness are based on a set of seemingly paradoxical structures. This article discusses the underlying paradox by disentangling it into five dialectical positions - activity vs. passivity, wanting vs. non-wanting, changing vs. non-changing, non-judging vs. non-reacting, and active acceptance vs. passive acceptance, respectively. Finally, the practical implications for the medical professional as well as potential caveats are discussed.

## Background

In the last two to three decades, the concept of mindfulness has received increasing attention, particularly in the health sciences. Mindfulness is about being aware of actual experiences from one moment to the next with gentle acceptance [1-3]. This concept has been proposed to contribute to the coping and recovery process in many health conditions.

Both clinical as well as basic science researchers have devoted a significant amount of study to this topic [4]. Moreover, with rapidly mounting evidence regarding the therapeutic capacities of mindfulness practice, medical professionals are increasingly incorporating such techniques into their clinical repertoire. Probably the best known and evaluated mindfulness-based treatment is the Mindfulness-Based Stress Reduction (MBSR) that is used in many clinical settings in the US and Canada and evermore, in Europe [4].

Yet, integrating mindfulness into existing therapeutic concepts may challenge medical professionals' usual practices for number of reasons. First and foremost, mindfulness approaches do not aim at symptom reduction. Fundamentally, mindfulness is not intended to explicitly eradicate pain, distress, or unwanted emotions. However, philosophically and practically, medical professionals endeavor to reduce suffering. If mindfulness does not aim at reducing symptoms, then how can it be helpful? In this essay, we argue that while mindfulness is not meant to actively reduce symptoms, it may passively modify their impact by changing an individual's perceptions and mindset. Mindfulness is a set of practices, if not a "way of being" that may incur salutogenic (i.e., health-promoting) effects. This may lead to a misconception of what mindfulness is, and how it works. We believe that some of the apparently contradictory aspects of mindfulness can be best understood by taking a dialectical approach. It is not a new idea to explain psychological health-related processes through the use of paradoxical or dialectical approaches [5]. Indeed, we propose that the dialectical structure of mindfulness hallmarks its essence, which may easily be misunderstood in clinical practice.

The dialectical approach is quite different from the conventional approach of symptom evaluation. The conventional approach uses the current logic: a symptom is either good or bad; present or absent; relevant or not. The dialectical approach stresses that each thesis also has to be considered in the light of its opposite (the antithesis), and only both facets together (the synthesis) yield a full picture. In this light, depression might be a sign of a disorder that should be mitigated. But at the same time, it must be acknowledged that there are inner experiences that cannot be controlled or altered "at will". Hence, although the phenomenal quality of going through depression may not be altered, a patient's *relation *towards relevant inner states relevant to depression may be changed due to mindfulness or other forms of spiritual exercise [6,7].

Herein, we first elaborate on the dialectical structure of mindfulness by providing an overview of 1) the theoretical foundation of the construct, 2) evidence of the clinical effectiveness, and 3) putative neurobiological correlates of mindfulness. We then introduce five dialectical positions that we believe are useful for resolving the apparent paradox associated with mindfulness and its relevant mechanisms of action. Finally, on the basis of this discussion, we derive the utility and implications of mindfulness for medicine, and address potential caveats.

### Roots of the concept of mindfulness

Mindfulness is an old concept; its theoretical roots were formulated by the Buddha, who characterized himself as a physician. He stated that his primary work was to identify the maladies afflicting humankind, and to establish a way through which every individual could attain lasting absence from suffering rather than complete well-being [8]. He claimed that mere belief and rational reasoning were not sufficient to mitigate suffering. He proposed mindfulness as a "direct way" to confront suffering by transcending it. Interestingly, the Buddha did not aim to establish an institutionalized form for disseminating his insights, such as a sect or a religion. His devotion to the self-reliance and self-dependence of each individual also demonstrates the very essence of mindfulness: "Do not believe in anything because it is rumored and spoken by many" [[9] p. 137]. He called for accepting only what one has analyzed by direct and immediate experience. On this basis, mindfulness can be described by its two inter-related facets: (1) the capacity to dispassionately observe the present moment, through (2) a stance of non-judgmental and accepting openness [10,11].

Unlike the contemporary practice of medicine that can be seen as "objective" and a mediated "third person perspective", mindfulness is experienced in the subjective and immediate "first person perspective", or by means of direct and unbiased introspection. Historically, this epistemological approach has also been part of the work of the psychologists Franz Brentano, William James, (and to some extent) Wilhelm Wundt and his disciple Edward Titchener, as well as by the phenomenologist Edmund Husserl [6,12]. The dialogical *I-Thou *philosophy of Martin Buber also evidences similarities to the concept of mindfulness [13].

Some scholars claim that the methodology of the science of inner experiences is not well developed [14,15] and that the phenomenological properties of inner experiences of consciousness cannot be shared. By contrast, the epistemological grounding of mindfulness - particularly to the Buddhist tradition - holds that great insight can be derived from first person approaches that are also valid from an intersubjective point of view [16]. For example, prolonged practice of mindfulness may develop the ability to dissect experiences into more subtle parts thereby revealing the transient nature of perceptions [17].

### Clinical effectiveness and neurobiological correlates of mindfulness

A body of research suggests that the facilitation of mindfulness has a positive impact on a variety of mental health symptoms, such as stress, anxiety, some personality disorders, chronic pain, and substance abuse. To date, several meta-analyses substantiate such clinical effects [1,18,19]. Additionally, there is evidence that mindfulness has an impact on endocrinological and neurophysiological function. For example, Davidson and colleagues found a greater increase in antibody titers after vaccination in individuals practicing mindfulness on a regular basis as compared to non-practitioners [20]. More recently, in a randomized trial, Tang et al. [21] found that mindfulness practitioners showed more regional cerebral blood flow in the right anterior cingulate cortex (ACC), including the subgenual ACC (Brodmann area (BA) 25), and adjacent ventral ACC (BA 32), the left insula, occipital lobule, right posterior cingulate cortex, right precuneus, and subcortical structures of the putamen and caudate. These brain areas have been related to emotional regulation [22]. Also, both Lazar et al. [23] and Hölzel et al. [24] demonstrated that mindfulness may be associated with an increase of gray matter which persists longitudinally in certain brain areas. These researchers found that gray matter concentration in the left inferior temporal gyrus was correlated with meditative proficiency, corroborating the assumption of a positive impact of meditation training on gray matter concentration in this region [25,26]. These results suggest that the ability of mindfulness to influence emotional state has not only a neurological base, but that mindfulness training - according to neuroplasticity paradigm - may actually influence the structural composition of the brain.

### Resolving the Paradox: The Dialectics of Mindfulness

In the following sections we discuss five aspects of mindfulness that are all related to the underlying dialectical principle. In our view, these five dialectical aspects are a valuable way to address the apparent paradoxical structure of the construct.

### Dialectics of Activity vs. Passivity

Mindfulness can be considered as a provokingly passive method. Mindfulness teachers explain the practice of mindfulness as "one simply examines every phenomenon" [[17] p. 95]. And, more illuminatingly, "the entire effort is to learn how not to react" [[17] p. 97]. Mindfulness implies calmly observing unwanted inner or outer experiences. Psychologically, this means that the drives related to appetitive or aversive stimuli must be voluntarily suspended to the most possible extent. The first drive has been termed "approach motivation" and the latter "avoidance motivation" [27]. Probably the most obvious difference to other treatments commonly employed, is that mindfulness advocates suspending cognitive, emotional or behavioral actions when facing negative experiences. In devising the antithesis, mindfulness seeks to engage a mechanism known as "apperception" a mental process that is responsible for perceiving, gauging, adopting and transforming an individual's experiences in order to create a new conscious concept based upon former experiences as well as inner states of the mind. Mindfulness involves meticulous and continuous observation of the prevalent sensory and mental processes by means of being fully present: Every moment and detail of a given experience is to be observed scrupulously, without cognitive and emotional evaluation of the respective event. This process involves a high degree of alertness and activity [28]. Hence, we claim that mindfulness is at the same time a way of being passive (with regard to gauging and reacting towards external stimuli) and of being active (with regard to observing the present moment and the accompanying inner states of mind). By means of reconfiguring the cognitively active and passive components, the thesis and antithesis of seemingly contradicting processes are resolved, and mindfulness can be regarded as a synthesis.

### Dialectics of Wanting vs. Non-wanting

Some authors argue that mindfulness is a "non-striving" state of being, thereby precluding goal-oriented behavior [29]. In other words, it is held that being mindful contradicts wanting, aspiring, or desiring something. These authors state that during a deep state of mindfulness, a person has no goals and does not want for anything during the time of practice. Although this concept may be appealing, we doubt that an individual can be in a state in which she does not want anything at all, at least in situations that entail active behavior. How could a person be motivated to begin and stay within a mindful state? The fact that an individual starts or continues a mindfulness exercise from one moment to the next explicitly shows that she *does *aspire to be mindful. Additionally, why should one engage mindfulness meditation at all? Would she not aspire to achieve a certain goal implicitly or explicitly associated with this technique? Hence, upholding mindfulness practice cannot be simply equated with "non-wanting", but rather implies wanting to achieve something such as reducing suffering and distress or gaining essential insights to phenomenological experience. At the same time, we admit that the mindfulness antithesis is at odds with a certain understanding of what "wanting" comprises. To be more precise, "unconditional wanting" is the opposite of mindfulness as it refers to a mental state where something is sought after to such a degree that not achieving the desired state would lead to dissatisfaction. It has been argued that unconditional wanting leads to breakdown of inner balance if the desired state is not achieved [17]. In fact, even if a person reaches the intended goal, the mind cannot be balanced, according to the theory of mindfulness, if the wanting is too strong. Both unconditional wanting or craving ("I must have this!") and aversion ("This must go away!") are forms of non-mindful states. Hence, the synthesis of the wanting-non-wanting dialectics reveals that a "gentle" form of wanting reflects the volitional processes underlying mindfulness, whereas "unconditional" wanting (both craving and aversion) cannot conform to the philosophy of mindfulness.

### Dialectics of Therapeutic Change vs. Non-Change

Medical professionals strive to alleviate suffering. Many medical professionals have used mindfulness techniques in order to lessen the suffering of the patients they treat [30]. Yet, mindfulness theory claims that pain and related symptoms must be accepted by embracing them with an attitude of equanimity. Accordingly, if this aspect of mindfulness is cultivated, there is no longer any real argument for changing inferior and negative sensations, emotions, or symptoms. This is a critical but often misunderstood aspect of mindfulness that rests upon the fact that changing one's relation to the symptom has been mistaken for changing the symptom. Empirical research indicates that mindfulness training leads to a reduction of pain and anxiety [31], not by directly lessening symptoms, but by changing patients' attitudes toward them. Hayes et al. [32] describe that mindfulness aims at establishing a psychological stance of preparedness in which formerly unwanted feelings can be accepted. To give an example, anxiety is not necessarily a problem, if anxiety is embraced with the right state of mind. Simply, if one is able to accept anxiety, anxiety gradually loses its effect, simply because it can be contained; finally, the degree of fear will decrease as a result of reduced emotional impact. Thus, mindfulness is about being aware of experience, and gently accepting it. The finding that mindfulness reduces psychosomatic symptoms may be explained by two mechanisms. First, if a symptom of distress, such as anxiety, is subjectively accepted and embraced rather than resisted, it becomes less threatening. This is likely the reason why decreased levels of distress, anxiety, and depression are reported after mindfulness interventions. Second, as such symptoms become less important, behavioral changes associated with decline of symptoms become more probable [33].

Recent research has shed light upon the possible mechanisms of mindfulness. Kohls, Sauer, and Walach described two main aspects of mindfulness, i.e., "presence" (attending to the present moment) and "acceptance" (non-judgmental attitude) [10]. Their work suggested that presence is a way to instill an attitude of acceptance, which is in turn responsible for buffering distress. It appears that presence itself does not buffer distress but allows one to build the non-judgmental attitude that seems to be responsible for health-relevant effects.

Taken together, the synthesis of change and no-change distinguishes between what is changed through the practice of mindfulness - an emotional connection with the symptom - and what is (primarily) unchanged - the symptom itself. As changes in the symptom itself are not intended, mindfulness may be understood as a transformation of the *relationship *between self and symptom.

### Dialectics of Non-judging vs. Non-Reacting

A facet of the definition of mindfulness is "non-judging", which has been advocated by Kabat-Zinn [34,35] and subsequently more widely accepted [29,36,37]. However, we suggest a word of caution here. Let us start by posing a question. Is it possible to cognitively perceive something and concomitantly to abstain from all judgments? To answer this question, it is necessary to understand judging. Literature on perception and cognitive psychology suggests that judging primarily entails identifying a perception as representative of a certain mental category (e.g., sparrow as a bird) [38]. The relevant category has different attributes including a valence continuum (from "I like it" to "I don't like it") with various degrees of intensity. It is unlikely that a well-learned category attribute ("weapons - not good") can be easily abandoned, if at all. We suggest that mindfulness in the first instance should not be equated with the *cognitive *aspect of non-judging.

Rather, we propose that mindfulness is primarily concerned with the *emotional-motivational *component of non-judging. Consider the following thought experiment. Imagine the possibility that you will not be eating your favorite food in the next months. This is not something agreeable; yet, although one would not want this to happen (i.e., cognitive judging occurs), it is possible to stay emotionally calm, and to remain contented despite this unwanted imagination (i.e., no emotional reaction). This emotionally calm state has been termed "balanced mind" - the fact that one does not react emotionally when facing an unfavorable experience [17]. The opposite behavior would imply generation of emotions of dislike or craving. As a result, an urge - a specific emotional-motivational tendency - to behaviorally react may arise. This reaction may entail either behavior to strengthen the favored experience, or weaken it, if it is disliked. Together, the emotional and motivational reactions become mutually reinforcing in a positive feedback loop. This may explain how such urges gain strength. Associated cognitions will likely accompany this process.

This is why a so-called unbalanced mind - primarily an emotional-motivational process - may lead to cognitions that are judging ("I hate this!"). This rationale provides some explanation why judging is incongruent with mindfulness, although judging is a secondary reaction or an epiphenomenon to mindfulness. Mindfulness is characterized by not showing reactive behavior or not indulging in emotions of craving and aversion. We do not argue for causal orders of evaluative cognitions and emotions. It may well be the case that specific cognitions exhibit associated emotions or vice versa [39-41]. Rather, our point is that a mindful state depends primarily on emotional calm, and only secondarily on the absence of evaluative cognitions. That means that one may calmly think "I don't like this at all" and still be mindful. Indeed, one cannot be upset and mindful at the same time.

### Dialectics of Active Acceptance vs. Passive Acceptance

Most authors in the field of mindfulness research consider "acceptance" as one of the major aspects of mindfulness, together with a form of directing the attention to the present moment. In this light, some, such as Kabat-Zinn, hold that mindfulness "includes an affectionate, compassionate quality" [[34] p. 145]. This conception may be interpreted as "active approval" of what is being experienced. This describes a stance of "the present experience is good" which includes emotional, motivational and evaluative aspects. But how can one approve *all *situations or experiences? This is seemingly illogical. In contrast to this assumption, we believe that mindfulness does not involve such "active" acceptance. We opine that the concept of "passive acceptance" is consistent with the essential idea of mindfulness, and simply suggests that one suspends or weakens evaluative cognitions and emotional reactions in a given situation. There is a subtle but crucial difference between the two concepts. The former assumes that one should actively strive to see the proverbial silver lining in all situations, although this may be contradictory to an individual's ethical or philosophical grounding. The latter view, in contrast, assumes that although we may not be able to escape a given situation, we may withhold our emotional or evaluative reactions, thereby possibly reducing the aversive character of a given situation. In other words, not accepting something does not necessarily imply rejecting it.

Thus, the difference between active and passive acceptance can be formalized by the concept(s) of passive and active negation [42]. To give an example, the active negation of "I love you" is "I hate you", whereas the passive negation is "I don't love you". In more formal terms, the active statement "A is p" may be negated by the active negation "A is (not p)" as well as by its passive negation "Not (A is p)". In sum, the synthesis suggests that mindfulness is the absence of reacting towards a given experience, but not the unconditional and active, rather naïve acceptance of a given experience.

## Conclusion

A number of practical conclusions may be drawn from the five forms of dialectics of mindfulness: (1) activity vs. passivity, (2) wanting vs. non-wanting, (3) changing vs. non-changing, (4) non-judging vs. non-reacting, and (5) active acceptance vs. passive acceptance, as presented in this paper. To begin with, individuals in a state of distress have a natural longing for suffering to end. Therefore, despite the well supported clinical efficiency of mindfulness treatments, it is crucial to explain to a patient that mindfulness is not a remedy such as anesthesia or analgesia. This is not to say that mindfulness is not intended to help - of course it is. But as it will help an individual "only" to live with the reality of a present moment, it should correspondingly be understood as a change in one's point of view, rather than a direct attempt to diminish a symptom. This is particularly relevant to the western medical system, given that modern medicine with all its successes and advantages has also fostered chronicity of certain illnesses that cannot be cured, and so must be cared for. Mindfulness may be a suitable avenue to that end.

Mindfulness is an approach that can be used to change reaction(s) toward unwanted experiences. Patients need to be aware of this point in order to avoid unrealistic expectations that may lead to disappointment before consenting to a mindfulness based intervention. To be more precise, a medical professional should be very clear when communicating to patients about what may *not *change (the symptom), and what *may *change (the relationship towards the symptom). As mindfulness practice may easily be misunderstood, "side effects" such as disappointment may occur as a result of having misinterpreted the concept.

Second, both theory and data corroborate that mindfulness is an experientially oriented approach. To become familiar with this different way of thinking, and in this way mobilize possible health benefits of mindfulness, it is necessary that patients practice regularly and actively. Accordingly, mindfulness interventions should not focus on theoretical discussions or explanations, but rather support active practice, although an initial orientation and repeated explanations may be necessary. In some ways, mindfulness is like swimming - it is best learned by doing. To date, conceptualizing along the lines of a dose-effect model, there are no valid conclusions regarding how much training is needed. However, most existing mindfulness interventions (such as the MBSR) work with rather high treatment schedule (e.g., 30 minutes homework per day and two hours group session per week) [34].

Third, the acceptance aspect of mindfulness should not be taken to the extreme. As stated above, it would be contrary to the concept of mindfulness (and also counterproductive) to simply embrace anything that happens with an accepting attitude. Rather, individuals exercising mindfulness on a regular basis should learn to voluntarily suspend the judging process as best possible. It is crucial to bear in mind the distinction between active and passive acceptance, as discussed above. It should also be stressed that mindfulness is not meant to be a "stand-alone" treatment. To the contrary, mindfulness approaches should be combined with more change-oriented approaches. Not doing so would entail the risk of providing suboptimal clinical intervention.

Patients should be encouraged to observe and register inner experiences without reacting to them. For this reason, some mindfulness schools teach student(s) to verbally express sensations. For example, in a case where the mindfulness student experiences a sensation of pain in the foot, the student would just state this perception- "there is a certain feeling of pain in the middle of my right foot"- without reacting to it.

The main caveat of working with mindfulness techniques is not to succumb to the escapist conception that one could "meditate the problem away by mindfulness". This would not be consistent with the nature of mindfulness. The problem is that clinicians (and perhaps humans, in general) are trained to think in cause-effect relations, try to identify the root of a problem, and then try to eliminate the cause. Without a cause, the problem should go away, and correspondingly the problem would seem to have vanished. This approach, although perfectly useful for survival in the external world, and despite having yielded tremendous progress in natural science and technology, may not work in the cognitive-emotional realm. Mindfulness training challenges the thought "if I get rid of my anxiety, I will live a fulfilled life" and replaces it with the statement "If I learn to accept my anxiety, I will eventually learn to live with it". This reflects the insight that although one cannot live a life without experiencing fear, she may be able to learn to master it. In other words, mindfulness may be a means by which one may be able to live a fulfilled life with a disorder by (passively) accepting it. Mindfulness should not be considered as a tool of cause-effect thinking. This is a difficult point, and it should be acknowledged that mindfulness involves a way of looking at the realities of the world that is different for much of a predominant paradigm of modern medicine.

In sum, mindfulness may prove to be an effective complementary approach that can be employed in a number of conditions to lessen subjective "illness". However, as we've shown, mindfulness differs substantially from the way that Western medicine approaches malady. Therefore, any medical professional who plans to incorporate mindfulness approaches into her therapeutic repertoire needs to recognize that it involves a dialectical, and not an "engineering"/curing, process. The dialectical character of mindfulness discussed in this essay is by no means complete; there are other aspects that may be worthwhile. Nevertheless, we believe that the five dialectical positions discussed - activity, wanting, change, judging, and acceptance - offer a promising starting point for understanding the construct(s) and process of mindfulness and its mechanisms of action.

In time, empirical evidence may elucidate in what circumstances, and to what extent mindfulness might be most useful within the therapeutic palette of clinical medicine. Our ongoing work is committed to this effort.

## Competing interests

The authors declare that they have no competing interests.

## Authors' contributions

SS wrote the initial draft. SL, HW and NK provided mentoring and extensive editing of the final manuscript. All authors read and approved the final manuscript.
